# Structure-based discovery of novel P-glycoprotein inhibitors targeting the nucleotide binding domains

**DOI:** 10.1038/s41598-023-48281-4

**Published:** 2023-12-01

**Authors:** Laust Moesgaard, Maria L. Pedersen, Carsten Uhd Nielsen, Jacob Kongsted

**Affiliations:** Department of Physics, Chemistry and Pharmacy, University of Southern Denmark, Odense M, 5230 Denmark

**Keywords:** Structure-based drug design, Virtual screening, Transporters

## Abstract

P-glycoprotein (P-gp), a membrane transport protein overexpressed in certain drug-resistant cancer cells, has been the target of numerous drug discovery projects aimed at overcoming drug resistance in cancer. Most characterized P-gp inhibitors bind at the large hydrophobic drug binding domain (DBD), but none have yet attained regulatory approval. In this study, we explored the potential of designing inhibitors that target the nucleotide binding domains (NBDs), by computationally screening a large library of 2.6 billion synthesizable molecules, using a combination of machine learning-guided molecular docking and molecular dynamics (MD). 14 of the computationally best-scoring molecules were subsequently tested for their ability to inhibit P-gp mediated calcein-AM efflux. In total, five diverse compounds exhibited inhibitory effects in the calcein-AM assay without displaying toxicity. The activity of these compounds was confirmed by their ability to decrease the verapamil-stimulated ATPase activity of P-gp in a subsequent assay. The discovery of these five novel P-gp inhibitors demonstrates the potential of in-silico screening in drug discovery and provides a new stepping point towards future potent P-gp inhibitors.

## Introduction

P-glycoprotein (P-gp), also known as ATP-binding cassette transporter B1 (ABCB1), is a transmembrane protein that belongs to the ATP-binding cassette (ABC) transporter superfamily. It is highly expressed in the liver, kidney, intestine, and endothelial cells of the blood-brain barrier (BBB), and plays a crucial role in cellular detoxification and efflux of various xenobiotics including drugs and toxins^1, 2^. This efflux activity makes it a major obstacle in drug discovery and development, as it can reduce the efficacy of many drugs by reducing their intracellular concentrations, leading to treatment failure or the development of drug resistance^3^. Therefore, P-gp has become an important drug target for overcoming multidrug resistance (MDR) in cancer chemotherapy and enhancing the bioavailability of drugs in many other therapeutic areas^4^.

**Figure 1:**
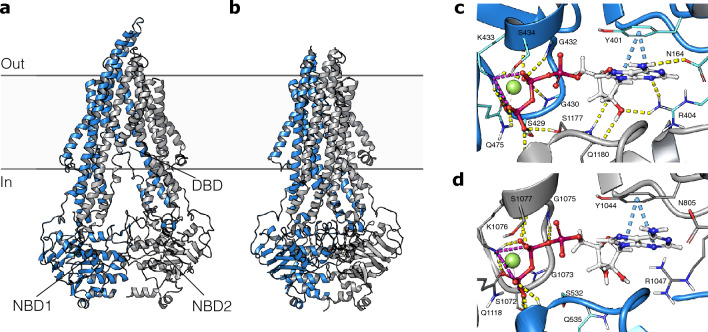
Structure of P-gp and the two nucleotide binding domains. (**a**) P-gp in the inward-open conformation (PDB ID: 6QEE). (**b**) P-gp in the outward-open conformation (PDB ID: 6C0V). (**c**) Structure of the nucleotide binding domain 1 (NBD1) and (**d**) structure of NBD2 with ATP bound. Yellow dashed lines: hydrogen bonds. Blue dashed lines: $\pi -\pi$ interactions. Purple dashed lines: salt bridges. Green spheres: magnesium ions.

P-gp is composed of two symmetric peptide chains that each has a nucleotide-binding domain (NBD) and a transmembrane domain (TMD) with six transmembrane helices (Fig. 1a,b)^5^. Substrate efflux is initiated by the binding of substrates at the large drug-binding domain (DBD) at the intracellular cleft between the two 12 transmembrane helices. The substrate is then pumped out of the cell as P-gp changes from an ‘inward-facing’ to a ‘outward-facing’ conformation in a peristaltic movement^6^. The change from the inward-facing to the outward-facing conformation is thought to be triggered by the binding of ATP, whereas the change back to the inward-facing conformation is powered by the hydrolysis of ATP^7^.

In previous studies, several inhibitors have been evaluated in clinical trials^8–10^. These previously discovered inhibitors primarily target the DBD, yet they tend to fall short of Lipinski’s rule of five (Ro5) compliance, as these inhibitors are generally large (molecular weight > 500 g/mol) and lipophilic (logP > 5). Another common characteristic among most inhibitors is their tendency to bind in a 2:1 stoichiometry^11–13^. These characteristics are to be expected, given that the substrate binding site has evolved to efficiently accommodate a wide array of hydrophobic molecules, resulting in its substantial size and hydrophobic nature^14^. Consequently, with the continuous need for safe and active inhibitors with limited side effects, it becomes imperative to explore alternative sites on P-gp that may offer more favorable drugability in the continued search for novel and improved inhibitors.

A second site that has been explored for its ability to inhibit the activity of P-gp is the nucleotide binding domains (NBDs) responsible for ATP binding and hydrolysis^15^. Numerous studies have demonstrated that various flavonoid derivatives can effectively inhibit P-gp by binding to these NBD sites^16–21^. As a number of these molecules resemble lead-like compounds in terms of size and polarity (MolWt < 460 g/mol, logP < 4), this suggests that it is indeed possible to design a novel generation of efficient P-gp inhibitors that block the NBDs. Furthermore, a previous structure-based study was able to identify molecules that show P-gp inhibition through binding at the NBDs and related sites, thus demonstrating the potential of targeting these remote sites^22–24^.

Recently, structure-based studies of large make-on-demand virtual libraries with hundreds of millions, and even billions of compounds have successfully led to the discovery of novel scaffolds with activities toward multiple different targets^25–28^. It has additionally been demonstrated that active learning techniques can be efficiently integrated to accelerate the screening process^29–31^. Therefore, to discover a new set of P-gp inhibitors, we screened a make-on-demand virtual library of 2.6 billion compounds by iteratively docking 6 million compounds in an active learning loop (Fig. S2). A diverse subset of the best-scoring molecules was then rescored using molecular dynamics (MD) simulations. Finally, 14 compounds from the refined subset were tested experimentally for their inhibition of P-gp mediated transport, leading to the discovery of 5 novel P-gp inhibitors.

**Figure 2:**
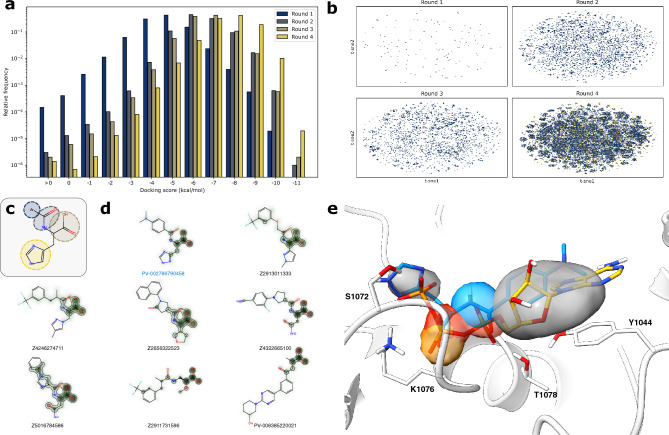
Results from the active-learning docking process. (**a**) Histogram of the distribution of docking scores from different rounds of the active-learning procedure. (**b**) t-sne visualization of the diversity of compounds found at different stages of the docking procedure with docking scores below -9 kcal/mol (top 0.01 % of docking scores). Visualization is based on dimensionality reduction of Morgan fingerprints of size 2048 and radius 2. (**c**) General representation of the scaffolds that most frequently scored well in the docking simulations. (**d**) Best-scoring compounds from the docking simulations belonging to different clusters. Atoms are shaded according to their impact on the machine-learning predictions. (**e**) Docking pose of PV-002786790458 compared to ATP. Surface indicates areas with high densities of different atom selections among top-scoring compounds. Grey surface: aromatic atoms, red surface: hydrogen bond acceptors, blue surface: hydrogen bond donors, and orange surface: carboxylic acids.

## Results and discussion

Initially, we aimed to obtain a well-defined structure of the nucleotide-binding domains (NBDs) in a ligand-bound state to serve as the starting point for our computational work. To achieve this, we focused on protein structures of nucleotides bound to the NBD of P-glycoprotein (P-gp). Since optimal ATP-binding to P-gp triggers a conformational change from the inward-open to the outward-open conformation, we selected a structure of ATP bound to an outward-open conformation of P-gp (PDB ID 6C0V) as our starting point. However, we hypothesized that inhibitors binding to the NBD may not necessarily induce the same conformational change. Therefore, we aimed to transform the outward-open structure into an inward-open state while preserving the ATP-bound shape of the NBD. To achieve this, we performed a targeted molecular dynamics (TMD) simulation of the transition from the outward-open to the inward-open conformation. This approach has previously been successfully applied in virtual screening for the discovery of NBD inhibitors of P-gp^22, 32^. To prioritize between the two different but highly homologous NBDs (Fig. 1c,d), a multitude of control-dockings using different MD snapshots were performed to evaluate the different site’s ability to distinguish known P-gp modulators from a set of property matched decoys (Table S2). Based on these calculations, the inward-open conformation of NBD2 was selected as the target structure in the consecutive large-scale docking.

In this study, we explored the lead-like subset of the diverse make-on-demand Enamine REAL space. The lead-like subset comprised 2.6 billion Ro5-compliant compounds at the time of the screen, with molecular weight less than 460 g/mol, logP between − 4 and 4, fewer than 4 rings, and less than 10 rotatable bonds. To efficiently navigate this vast chemical space, we employed an active learning protocol using a Graph Convolutional Network (GCN)^33^ to suggest molecules with good docking scores (Fig. S2). The GCN was able to predict docking scores of $\sim$ 5600 molecules per second on a single Nvidia GeForce RTX 3080 graphical processing unit (GPU), which is significantly faster than traditional brute-force docking methods (AutoDock GPU $\sim$ 5 molecules per second on the same setup). In total, we docked 6 million compounds from the Real Lead-Like library in three learning iterations with 1 million compounds in each and a final screen of 3 million compounds. This resulted in a diverse set of molecules with favorable docking scores (Fig. 2a,b). Through bootstrapping analysis applied to the initial random sample of 1 million compounds, we estimated that we found between 41 and 65% of all compounds with docking scores better than − 9 kcal/mol (Fig. S3). Notably, this was achieved despite docking less than 0.3 % of the entire library.

**Figure 3:**
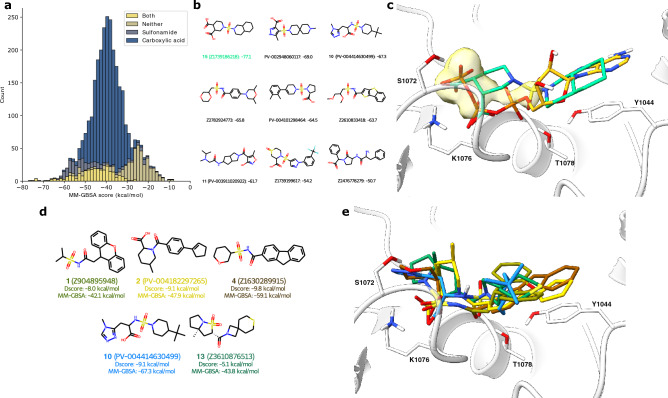
Results from the MM-GBSA screening process. (**a**) Histogram of the distribution of MM-GBSA scores grouped according to the molecules’ presence of carboxylic acids and sulfonamides. (**b**) Best-scoring molecules from nine different clusters. (**c**) Docking pose of **15** (Z1739186218) compared to ATP. Yellow surface indicates areas with high densities of SO$_{2}$-groups. (**d**) 2D-representation of active molecules with their docking score (Dscore), and MM-GBSA score (MM-GBSA). (**e**) Docking poses of all active compounds. Molecules are colored according to the labels in (**d**).

In the second and third rounds of docking, we applied a similarity filter to ensure some degree of exploration in these rounds of the active learning process, whereas a greedy selection was used for the final round of docking. As expected, this approach resulted in a significantly higher percentage of compounds with favorable docking scores in the final round compared to the previous rounds (Fig. 1a). However, we also observed far less scaffold exploration in the final round (Fig. S5). Interestingly, we found significant overlap between the compounds from the second and third rounds in the parts of the chemical space that they explore, indicating that the machine learning model had already largely converged at this stage of the active learning procedure (Fig. 2b).

Through a closer examination of the compounds with the best docking scores, it was found that nearly all of them contained a carboxylic acid group adjacent to an amide group (Figs. 2c, S5). These findings suggest that many of the synthons used to construct these molecules are amino acids. Interestingly, it was observed that the peptide functionality was typically surrounded by two aromatic ring structures, with the ring closest to the carboxylic acid being predominantly five-membered. To gain insight into the key factors affecting the deep learning model’s prediction of a molecule’s docking score, an atom removal approach was applied to estimate the contributions of individual atoms to the predicted scores (Fig. 2d)^34^. The results showed that the peptide functionality of the best-scoring compounds contributed the most to the predicted docking scores, which explains why these substructures were very commonly found among the best-scoring molecules.

To achieve a better understanding of the importance of specific substructures in our study, we investigated the grid densities of these substructures within the NBD and visualized them (Fig. 2e). Our findings revealed that the carboxylic acid was frequently situated at the position around the ATP beta-phosphate in the reference structure. This position is at the center of a Walker A motif, a common motif for binding the beta-phosphate of ATP. As carboxylic acids resemble the phosphate groups of ATP since they at neutral pH have a negative charge of one stabilized by multiple oxygens, this observation was not unexpected. Furthermore, the hydrogen bond acceptor density of the molecules’ amide carbonyl was closely aligned with the position of the alpha-phosphate of ATP, which allowed for the formation of hydrogen bonds to T1078 and the backbone of S1077. Consistent with expectations, a large aromatic density was located at the nucleobase binding site, while a smaller density was found at the gamma-phosphate binding region. In general, the best-scoring molecules seemed to share a significant number of features with ATP, which exemplifies how well the binding site has adapted to bind ATP.

As docking uses a rigid representation of the binding site, we wanted to explore the effects of introducing flexibility. We, therefore, performed multiple series of MD simulations to calculate MM-GBSA scores for a diverse set of compounds selected based on their docking score, molecular properties, diversity, and machine learning predicted MM-GBSA scores. As expected, since the MD simulations use the docking poses as the starting point, we found that the MM-GBSA scores and docking scores were generally correlated (Pearson correlation coefficient = 0.42, Fig. S7). Surprisingly, we observed that compounds with higher docking scores (− 9.2 kcal/mol) on average outperformed those with lower docking scores (− 9.6 kcal/mol), suggesting a discrepancy between the functionalities preferred by MM-GBSA and Glide. Upon examining the molecules with good MM-GBSA scores, we found that the best-scoring molecules in docking predominantly had a carboxylic acid group, while the best-scoring MM-GBSA molecules instead contained a sulfonamide group. In fact, the overwhelming majority of compounds that achieved good MM-GBSA scores had at least a sulfonamide or a carboxylic acid and often both (Fig. 3a,b). By visualizing the density of the sulphone among the best-scoring compounds in docking, we observed a high density around the beta-, and gamma-phosphate of ATP, while only a smaller density was observed at the alpha-phosphate position (Fig. 3c). These computational findings suggest that the sulphone group is as good a computational isostere as a carboxylic acid for the phosphates of ATP.

Based on the findings from docking and the MM-GBSA calculations, a set of 14 molecules was selected for experimental testing (Fig. S8). The molecules were manually selected to reflect the molecular diversity and interaction diversity of the best-scoring molecules in docking and MM-GBSA. The selected compounds were tested for their ability to inhibit P-gp mediated transport using two different assays, i.e., the calcein-AM cellular export in intact MDCKII MDR1 cells with quinidine as a control and inhibition of verapamil induced ATP consumption in Sf9 insect cell vesicle expressing human MDR1 using tariquidar as a control.

Initially, the compounds were investigated for the ability to concentration-dependently inhibit calcein-AM efflux in MDCKII MDR1 cells at extracellular pH values of 7.4 and pH 5.5 (Figs. S9, S10). Notably, at an extracellular pH of 7.4, 5 of 14 investigated compounds exhibited more than 50% inhibition of calcein-AM efflux at the highest concentration investigated. Hence, IC50 values were estimated, and these are shown in Table 1. Following the calcein-AM assay, cellular toxicity was investigated, revealing that none of the compounds tested at pH 7.4 affected MDCKII MDR1 cell viability (Fig. S11). To investigate if extracellular pH affected the ability of the compounds to diffuse to and bind to the NBD of P-gp, the calcein-AM assay was also performed at pH 5.5 (Fig. S11). Notably, altering the extracellular pH yielded results largely consistent with those observed at pH 7.4, while still not showing signs of cellular toxicity. This finding suggests that the negative charge on the acidic compounds does not strongly impair the compounds’ ability to permeate the cell membrane.

The compounds showing an ability to inhibit calcein-AM efflux at both pH 5.5 and pH 7.4, i.e. **1**, **2**, **4**, **10**, **13**, were subsequently investigated for their ability to inhibit the P-gp mediated consumption of ATP stimulated via substrate transport of verapamil. As shown in Fig. S11A, verapamil stimulated sensitive P-gp ATPase activity, and this activity could be reduced to the background buffer level through incubation with the P-gp inhibitor tariquidar, used as a control inhibitor (Fig. S12A). The inhibition of P-gp by tariquidar was confirmed by concentration-dependent inhibition (Fig. S12B), thereby validating the setup with Sf9 vesicles and UV-analysis. The ability to inhibit sensitive P-gp ATPase activity stimulated via substrate transport of verapamil by **1**, **2**, **4**, **10**, **13** was subsequently investigated (Fig. S12C and Table 1). The investigated compounds were relatively similar in their ability to interact with the NBD of P-gp, as the IC50 values determined range from 332 to 1300 µM. In comparison, the binding affinity for ATP to P-gp has been reported to be in the range of 300–800 µM^35–38^. Although IC50 values and binding affinities are not directly comparable, the observations indicate that the interaction between the compounds and the ATP bindings sites are not majorly different from ATP itself. For the tested compounds, we did not observe any clear correlation between either docking score or MM-GBSA score and activity (Fig. S13).

**Table 1:** Affinity values for investigated compounds in 3–4 individual cell passages (n = 3–4), except for quinidine which was investigated in n = 11–12.

	ATP assay			Calcein-AM, pH 7.4			Calcein-AM, pH 5.5		
Paper ID	IC50 (µM)	Log IC50	± SE	IC50 (µM)	Log IC50	± SE	IC50 (µM)	LogIC50	± SE
1	1300	3.11	0.34	324	2.51	0.14	390	2.59	0.67
2	607	2.78	0.26	719	2.86	0.37	760	2.88	0.44
4	1030	3.01	0.24	605	2.78	0.28	1033	3.01	0.69
10	332	2.52	0.15	299	2.48	0.27	127	2.10	0.25
13	590	2.77	0.23	78	1.89	0.16	44	1.65	0.19
Tariquidar	0.11	− 0.96	0.22						
Quinidine				1.89	0.28	0.09	27.49	1.44	0.11

In examining the structure-activity relationship of compounds with measurable activity as P-gp inhibitors, we found that five molecules shared similar features, including carboxylic acids, amides, and sulphonamides located in the middle of the molecule (Fig. 3d). When comparing the docking poses for these compounds to the docking poses of the inactive molecules, we identified three common features (Fig. 3e): (1) a carboxylic acid or a sulphone positioned inside the Walker A loop, which is equivalent to the beta-phosphate position of ATP; (2) at least one hydrogen bond acceptor, either a carbonyl or a sulphone, in the position of the alpha-phosphate of ATP; and (3) a hydrophobic part corresponding to the sugar/nucleobase part of ATP. We observed significant variation in the structure of the hydrophobic part of the molecules, suggesting that modifications can be made to improve inhibitor potency. Interestingly, one of the molecules that showed experimental activity, **15**, matches the best-predicted scaffold discovered in docking, which supports the validity of the docking model. Despite the fact that most of the molecules were negatively charged at physiological pH, one compound, **13**, surprisingly displayed activity despite not having a negative charge. These findings provide a valuable starting point for the design of future NBD inhibitors of P-gp with different scaffolds from the currently known flavone derivatives.

## Conclusion

In this study, we used an active learning protocol to virtually screen the Enamine Real space to identify novel inhibitors of the nucleotide binding domain (NBD) of P-gp. To supplement the docking scores, we performed a series of MD-based MM-GBSA calculations on 3015 compounds, which along with docking led to the discovery of specific scaffolds with good predicted binding affinity towards the NBD of P-gp. 14 compounds were selected based on their predicted properties and tested experimentally for their activity towards P-gp. Five diverse compounds showed biological activity, which provides valuable information for future molecular design of novel inhibitors for the NBD of P-gp.

## Material and methods

### Preparation of system for molecular dynamics simulations

The closed conformation of P-gp was acquired from the Protein Data Bank (PDB id: 6C0V, resolution: 3.4 Å)^39^. Missing loops of the protein was added using the SWISS-model homology modelling server^40^, and the full structure was imported into the Maestro module, which is available with the Schrödinger Suite^41^. The Protein Preparation Wizard module in Maestro was used to add and minimize bonded hydrogen atoms and to determine protonation states using Epik^42^. The prepared protein structure was then exported and uploaded to the Charmm-Gui Membrane Builder, to embed the protein in a lipid bilayer^43, 44^. The bilayer was composed of 120 POPC and 30 cholesterol lipids in the upper leaflet, while the lower leaflet held 117 POPC 29 cholesterols lipids. The membrane-protein system was solvated and neutralized in a 0.150 M KCl waterbox, which contained 45066 water molecules. The solvated system was exported and an amber topology file was built using tleap, which is available with amber^45^ using the lipid17 parameters (lipids)^46^, the ff19SB parameters (protein)^47^, the TIP3P parameters (water)^48^ and polyphosphate parameters (ATP)^49^.

### Targeted molecular dynamics simulation

MD simulations were performed using NAMD2^50^ with amber settings, with a cutoff of 10 Å, with the SHAKE algorithm applied, and using the Particle Mesh Ewald (PME) method. An initial minimization of 10,000 steps was performed without constraints to remove bad contacts. The protein backbone and solvent atoms were then fixed while a short minimization of 2000 steps and a 100 ps simulation at 303 K were performed to melt the lipid bilayer. The simulation was performed with a timestep of 1 fs under the NVT ensemble controlled using Langevin dynamics. The constraints were then removed from the solvent and 150 ps of simulation under the NPT ensemble with a timestep of 1.5 fs was performed. The pressure was controlled using the Berendsen barostat, while the Langevin thermostat was continuously used. A 10 ns equilibration simulation of the system was then performed using the same settings but with a 2 fs timestep and no constraints on the backbone. This was followed by a 50 ns targeted MD (TMD) simulation, which was performed using an aligned structure of P-gp in the open conformation as reference (PDB id: 6QEE, resolution 3.9 Å)^51^. The biased simulation was performed with an elastic constant of 200 kcal/mol/Å$^{2}$ with a 2 fs timestep. Finally, the system was simulated for 50 ns in the open conformation without any restraints. Root mean square deviation (RMSD) of the nucleotide binding domains and the backbone was used to guide the selection of snapshots from the MD simulations with different conformations of the binding sites (Fig. S1). Snapshots after 0, 8, 20, 30, 50, and 100 ns were extracted from the last 100 ns of simulation for docking studies.

### Test set for docking enrichment

A dataset with *Active* and *Marginal* inhibitors was collected from literature, based on their ability to inhibit P-gp through binding to the NBDs (Table S1). These were used to make two datasets: one containing only the compounds characterized as *Active* based on their high activity, and one which also contained compounds with poorer activity. Both datasets were used to create test sets for docking enrichment by adding decoy compounds, which were generated using the DUD-E server^52^.

### Preparation of docking grid

The six extracted snapshots from the MD simulations were imported into the Maestro module and minimized using the Protein Preparation Wizard^41^. Four grids were generated for each of the snapshots: one grid for each of the NBDs with and without magnesium present. The grids were defined by boxes centered at the center of geometry of ATP. Three docking experiments were performed on each of the 24 grids: one re-docking ATP and one for each of the two test sets. The grids were all evaluated based on the RMSD of the ATP docking pose compared to the original pose and on ROC/RIE scores of the test sets (Table S2). Based on these results, the grid built from NBD2 in the snapshot after 50 ns of simulation (the final frame of the TMD simulation) without magnesium bound was selected for high throughput virtual screening (HTVS).

### Active learning accelerated docking

A workflow diagram of the active learning procedure can be found in Supplemental Information (Fig. S2). The Enamine REAL lead-like compound database, which at the time of download contained 2.654 billion compounds, was retrieved from the Enamine website. A random sample of one million compounds was then extracted from the database. Protonation states at pH 7 ± 1.0 and stereoisomers for all compounds in the sample were generated using LigPrep, which is available with the Maestro Suite. The prepared sample was then docked to the previously generated grid using SP Glide^53^. The best-achieved docking score for each compound was extracted and used to train a graph convolutional network (GCN) as described elsewhere^33, 54^. More specifically, the regression model was trained to predict the docking scores of the scored molecules, which were represented as graphs. The Pearson R$^{2}$ score was used as the scoring metric, and training was performed with a batch size of 128. The GNN was then used to make predictions on the entire lead-like database and, based on this prediction, the best 100 million compounds were extracted. A diverse sample of one million compounds was then selected from the 100 million compound subset and used for another round of docking. The molecular similarity was determined using RDKit^55^. Docking results from both iterations were then used to retrain the GNN and generate a new 100 million compound subset. Once again, the selection, docking, GCNN training, and prediction processes were repeated, and the best 3 million compounds were extracted for a final round of docking. In total, 6 million compounds were docked.

### MM-GBSA protocol

MD simulations of seven random compounds with docking scores below − 10 kcal/mole, were performed to determine an optimal simulation protocol for the convergence of MM-GBSA calculations. The compounds were inserted into NBD2 of P-gp at the endpoint of the TMD simulation by utilizing their docking poses and removing ATP and magnesium. Generalized amber force-field (GAFF) parameter files for the ligands were generated using the AM1-BCC charge method in Antechamber, which is available with amber^56–59^. Simulations were performed using Amber software, using the SHAKE algorithm to constrain bonds involving hydrogens, and using a timestep of 2 fs^45, 60^. Initially, 500 steps of minimization using the steepest descent algorithm were performed followed by 500 steps using gradient descent. The systems were then heated to 300 K through 10 ps of simulation applying Langevin dynamics with constant volume and with restraints on the protein backbone and the ligands. This was followed by 10 ps of NTP simulations using Berendsen anisotropic pressure regulation while retaining the restraints. Finally, restraints were lifted and 200 ps of NTP simulation was performed while writing coordinates of the system every 5 ps. The simulation process was repeated 50 times for each ligand using an electrostatic cutoff of 12 Å and then again using a cutoff of 8 Å. The mmpbsa.py program was used to calculate the Molecular Mechanics Generalized Born Surface Area (MM-GBSA) score between the ligands and P-gp at every frame from the final 200 ps of each simulation. Based on these results, it was determined that 10 replica simulations with a production length of 80 ps and with a cutoff of 8 Å was sufficient to reach converged MM-GBSA scores (standard error of mean < 0.6 kcal/mole, Fig. S2).

### MM-GBSA calculations

Of the six million docked compounds, 180,704 were found to have a docking score of less than − 9 kcal/mole. RDKit was used to determine a subset of these molecules with less than 0.4 Tanimoto similarities to other compounds within the subset, totaling 1117 compounds. An MM-GBSA score was determined for each of these compounds using the protocol described above. The MM-GBSA calculations had a completion rate of 97.6%. The MM-GBSA scores were used to finetune the GROVER$_{large}$ model for 100 epochs^61^. The model was then used to reevaluate all compounds with docking score $< -9$ kcal/mole and extract the best 1500 compounds. Of these compounds, a new set of 200 compounds was extracted with minimal internal similarity and minimal similarity to the previously tested. Simulations and MM-GBSA calculations were then performed for these compounds before the ML model was finetuned again and a new set of compounds were selected and tested. This ML loop was repeated until a dataset of $\sim$ 1700 MM-GBSA scores were collected. After another finetuning of the ML model, the 4000 compounds with the best-predicted score were extracted. From this subset, 800 compounds with less than two acidic groups were extracted for MM-GBSA scoring based on diversity. Finally, the model passed through a final round of training before it was used to predict MM-GBSA scores on all docked compounds with a docking score $< -4$ kcal/mole. Based on this prediction and molecular properties such as charge, mass, number of rotatable bonds, and diversity, 522 compounds were selected for a final round of MM-GBSA scoring. In total, 3015 compounds were successfully scored using the MM-GBSA protocol.

### Materials

Test compounds, see Table S3, were purchased from Enamine (Kyiv, Ukraine). MDCKII MDR1 cells were obtained from the Netherlands Cancer Institute (Amsterdam, the Netherlands). ABC transporter Vesicles contained purified plasma membranes isolated from Sf9 cells infected with baculovirus expressing human P-gp were from GenoMembrane Co., Ltd (Kanagawa, Japan). Black 96-well microplates with clear bottom were from Corning Life Sciences and purchased through Merck (Readington Township, NJ, USA). Dimethyl sulfoxide (DMSO), calcein acetoxymethyl ester (calcein-AM), quinidine, verapamil, 4-(2-hydroxyethyl)-1-piperazineethanesulfonic acid (HEPES) and 2-(N-morpholino)ethanesulfonic acid (MES) were at purities of $\ge$ 95% and Dulbecco’s modified Eagle medium (DMEM), penicillin/streptomycin (100 ×), L-glutamine (200 mM), non-essential amino acids (1%), sodium bicarbonate solution (7.5%), Trypsin-EDTA (10 ×) suitable for cell culture and sterile-filtered, Tris (hydroxymethyl) aminomethane HCl (Tris), 3-(N-Morpholino) propanesulfonic acid (MOPS), potassium chloride, magnesium chloride hexahydrate, KCl, NaN$_{3}$, sodium dihydrogen phosphate dihydrate, sodium dihydrogen phosphate dihydrate, sodium orthovanadate, Na$_{2}$ATP, MgCl$_{2}$, sodium dodecyl sulfate (SDS), zinc acetate, mannitol, aprotinin, leupeptin were from Merck (Readington Township, NJ, USA) and were applied in the received quality, which was analytical-grade or higher. Zosuquidar was from MedKoo Biosciences Inc. (Morrisville, NC, USA). Hanks Balanced Salt Solution (HBSS) (10 $\times$) buffer was suitable for cell culture, sterile-filtered, and ascorbic acid, sodium azide, ouabain, zinc acetate dihydrate, ammonium molybdate and dithiothreitol (DTT) were all purchased from Gibco through Thermo Fisher Scientific (Waltham, Massachusetts, USA). Fetal Bovine Serum (FBS) was from Biowest (Kansas City, PA, USA) and EGTA was from VWR (Radnor, Pennsylvania, USA). CellTiter-Glo Luminescent Cell Viability Assay was from Promega (Madison, Wisconsin, USA). A water purification system was applied to obtain ultrapure water (Millipore, Boston, MA, USA).

### Cell culture

MDCKII MDR1 cells were sub-cultured as previously described^62^ and seeded on black 96-well microplates (clear bottom) at 3.2 $\times$ 105 cells/ml corresponding to 200,000 cells/cm$^{2}$ and used three days (72 h) after seeding. The cells were maintained in an incubator at 37 $^{\circ }$C in a humidified atmosphere supplemented with 5% CO$_{2}$. The culture medium was changed every 2–3 days and consisted of DMEM supplied with 10% FBS, penicillin/streptomycin (10,000 U ml$^{-1}$/10 mg ml$^{-1}$), L-glutamine (1%), and non-essential amino acids (1%).

### Preparation of compound solutions

Test compounds were used as received with no further purification. Initially, the compounds were dissolved in DMSO to the highest possible concentration, see Table S3. For the calcein-AM and cell titer viability assays, working solutions were made by diluting the DMSO stocks with HBSS supplemented with 10 mM HEPES adjusted to pH 7.4 ± 0.05 (HBSS$^{7.4+}$) or HBSS supplemented with 10 mM MES adjusted to pH 5.5 ± 0.05 (HBSS$^{5.5+}$). In order to ensure cell compatibility, the highest concentration of compound used in the assay contained 1% DMSO. For experiments with P-gp expressing vesicles, the DMSO stock solutions were diluted with the reaction buffer consisting of 50 mM MOPS-Tris, 50 mM KCl, 0.1 mM EGTA, 5 mM NaN$_{3}$, 2 mM DTT, and 1 mM Ouabain to a final highest concentration of 2% DMSO. All solutions used in the vesicle assay contained 2% DMSO.

### Calcein-AM efflux assay

Calcein-AM efflux experiments were performed on MDCKII MDR1 cells, which were seeded on black 96-well microplates. Before the experiments, media was removed from the wells and 50 µL pre-warmed HBSS$^{7.4+}$ was added. After a 15 min incubation period, the HBSS$^{7.4+}$ buffer was removed and 50 µL of solution containing P-gp inhibitor or test compound dissolved in HBSS$^{7.4+}$ or HBSS$^{5.5+}$ were added to the individual wells, followed by addition of 50 µL 10.0 µM calcein-AM giving a final calcein-AM concentration of 5.0 µM. Calcein-AM diffuses into the cell where it is either hydrolysed to a membrane-impermeable fluorescent calcein dye or extruded from the cell by P-gp^63^. Inhibiting P-gp increases the amount of calcein in the cell and a higher amount is hydrolysed to calcein, thus the fluorescence measured is increased. The highest concentration of each compound was added to a cell-containing well in the absence of calcein-AM to investigate if compounds were fluorescent themselves. None of the compounds investigated showed any fluorescence at the wave lengths used. Wells were excited at 485 nm and emission was measured at 520 nm. The fluorescence at 520 nm was recorded using enhanced dynamics range for 30–60 cycles (60 s cycle$^{-1}$) at 37 $^{\circ }$C using a CLARIOstar Plus Microplate Reader from BMG LABTECH (Ortenberg, Germany).

### ATP-consumption in P-gp expression vesicles

P-gp-mediated drug transport across membranes is driven by energy derived from hydrolysis of ATP. It is, therefore, possible to evaluate drug binding to P-gp by determining the ATPase activity. The method was modified from the procedure reported by Sarkadi et al.^64^. Briefly, solutions of test compounds and positive and negative controls in a reaction medium containing 50 mM MOPS-Tris (pH 7.4), 0.1 mM EGTA, 2 mM DDT, 50 mM KCl, 5 mM sodium azide, and 1 mM ouabain were incubated on ice in a 96 well plate. Then P-gp containing membrane vesicles to a final concentration of 0.5 mg/ml were added on ice to each well. The reaction medium containing a final verapamil concentration of 50 µM was added to the wells and the wells were gently mixed and incubated at 37 $^{\circ }$C for 3 min shielded from the light. Then, the ATPase reaction was started by the addition of MgATP to a final concentration of 5 mM and the plates with vesicles were incubated at 37 $^{\circ }$C for 60 min. The reaction was stopped by the addition of a 10% SDS solution and the amount of inorganic phosphate was determined immediately by addition of a coloring solution containing final concentrations of 8 mM ascorbic acid, 10 mM zinc acetate, and 7 mM ammonium molybdate. The wells were then incubated at 37 $^{\circ }$C for 20 min and the absorbance was scanned at wave lengths from 630 to 850 nm, and 740 nm was used for calculation of ATP consumption based on a phosphate standard curve freshly made with each experiment. To estimate sensitive ATPase activity mediated by P-gp, all experiments were conducted with no compound controls (2% DMSO), positive controls and background controls in which no stop solution was added, all in the absence and presence of 500 µM vanadate. The absorbance measured for a test condition was converted to nmol inorganic phosphate and normalized for reaction time and protein amount. The sensitive ATPase activity was then obtained by subtracting the inorganic phosphate generated in the presence of 500 µM vanadate at otherwise similar conditions.

### Cell viability assay

The viability of the cells was investigated using the CellTiter-Glo^®^ assay measuring cellular ATP levels using the solutions described above for the calcein-AM assay and a 1-h incubation. For the CellTiter-Glo^®^ assay, the growth medium was aspirated. 50 µL test compound was added to the wells and incubated for 1 h at 220 rpm and 37 $^{\circ }$C. During the experiments, plates were protected from light with aluminum foil. For measurement of ATP, the CellTiter-Glo^®^ Reagent (CellTiter-Glo^®^ Buffer and CellTiter-Glo^®^ Substrate) was thawed and equilibrated to room temperature and mixed prior to use. The cell-containing plate was after 1 h incubation equilibrated for 10 min at room temperature buffers were removed and replaced by 50 µL HBSS$^{7.4+}$ or HBSS$^{5.5+}$ buffer and 30 µL of CellTiter-Glo^®^ 2.0 Reagent was added directly to the wells. The plate was mixed on an orbital shaker for 2 min at room temperature. The plate was then incubated at room temperature for 10 min to stabilize the luminescent signal. The viability was assessed by normalizing the luminescence recorded in the presence of a given concentration of a compound to the luminescence obtained in control cells incubated in HBSS$^{7.4+}$ or HBSS$^{5.5+}$ buffer only.

### Data analysis

Calcein fluorescence in arbitrary unit (a.u) corrected for background signal, which was the signal obtained from the cells incubated with HBSS$^{7.4+}$ or HBSS$^{5.5+}$ without calcein-AM, was plotted as a function of time and slopes (denoted m below) were obtained by linear regression. The increase in fluorescent calcein is a surrogate marker of increased P-gp inhibition^63^. The slope from curves of fluorescence as a function of time for a compound ($m_{compound}$) was normalized to the slope of fluorescence in cells exposed to HBSS$^{7.4+}$ or HBSS$^{5.5+}$ only (referred as control, $m_{control}$). In the present work the inverse relationship of the normalized response was obtained as a calcein-AM efflux relative to control using Eq. (1):

1 $$\begin{aligned} \text {Calcein-AM efflux} = \text {P-gp activity} = \left( 1 \div \left[ \frac{m_{compound}}{m_{control}} \right] \right) \end{aligned}$$

The calcein-AM efflux or sensitive ATPase activity was plotted as a function of logarithmic concentrations of compounds, and IC50 values were obtained by fitting the data to nonlinear regression using the three-parameter equation in GraphPad Prism 9.3.1 (see Eq. 2).

2 $$\begin{aligned} Y = \frac{\text {Bottom} + (\text {Top}-\text {Bottom)}}{1+10^{X-\text {LogIC50}}} \end{aligned}$$

### Statistics

Data were obtained from at least three independent passages or independent vesicle experiments (n = 3). The results are presented as mean values ± SEM and statistical analysis was performed by utilizing GraphPad Prism 9.3.1.

### Supplementary Information

Supplementary Information.

## Data Availability

All data generated or analysed during this study are included in this published article and its supplementary information files. Requests for material should be made to the corresponding authors.
