# Supplementary material for: Structure-based discovery of novel P-glycoprotein inhibitors targeting the nucleotide binding domains
Source: Sci Rep. 2023 Dec 1;13:21217. doi: 10.1038/s41598-023-48281-4 (PMC10692163; doi:10.1038/s41598-023-48281-4)
Supplement: Supplementary file 1 — Supplementary Information. [file 41598_2023_48281_MOESM1_ESM.pdf]

Structure-based discovery of novel P-glycoprotein inhibitors targeting the nucleotide binding domains

Laust Moesgaard*, Maria L. Pedersen, Carsten Uhd Nielsen, Jacob Kongsted
Department of Physics, Chemistry and Pharmacy, University of Southern Denmark, Odense M, DK–5230, Denmark
*moesgaard@sdu.dk

November 1, 2023

Contents

1 Fig. S1: Targeted molecular dynamics data 2

2 Table S1: Known P-gp nucleotide binding domain inhibitors 3

3 Table S2: Results from retrospective dockings 13

4 Fig. S2: Active learning Workflow 14

5 Fig. S3: Running average of the MM-GBSA score for the test simulations of P-gp bound to different ligands. 15

6 Fig. S4: Bootstrap estimation of docking score distribution of the full dataset 16

7 Fig. S5: Number of molecules belonging to the most populated scaffold for each round of the docking procedure. 17

8 Fig. S6: Bemis-Murko scaffolds with best docking scores 18

9 Fig. S7: MM-GBSA results 19

10 Fig. S8: Experimentally tested compounds 20

11 Table S3: Overview of compounds and stock solutions 21

12 Fig. S9-11: Results from calcein-AM assay 22

13 Fig. S12: Results from ATPase assay 29

14 Fig. S13: Comparing docking and MM-GBSA in predicting activity 30

1 Fig. S1: Targeted molecular dynamics data

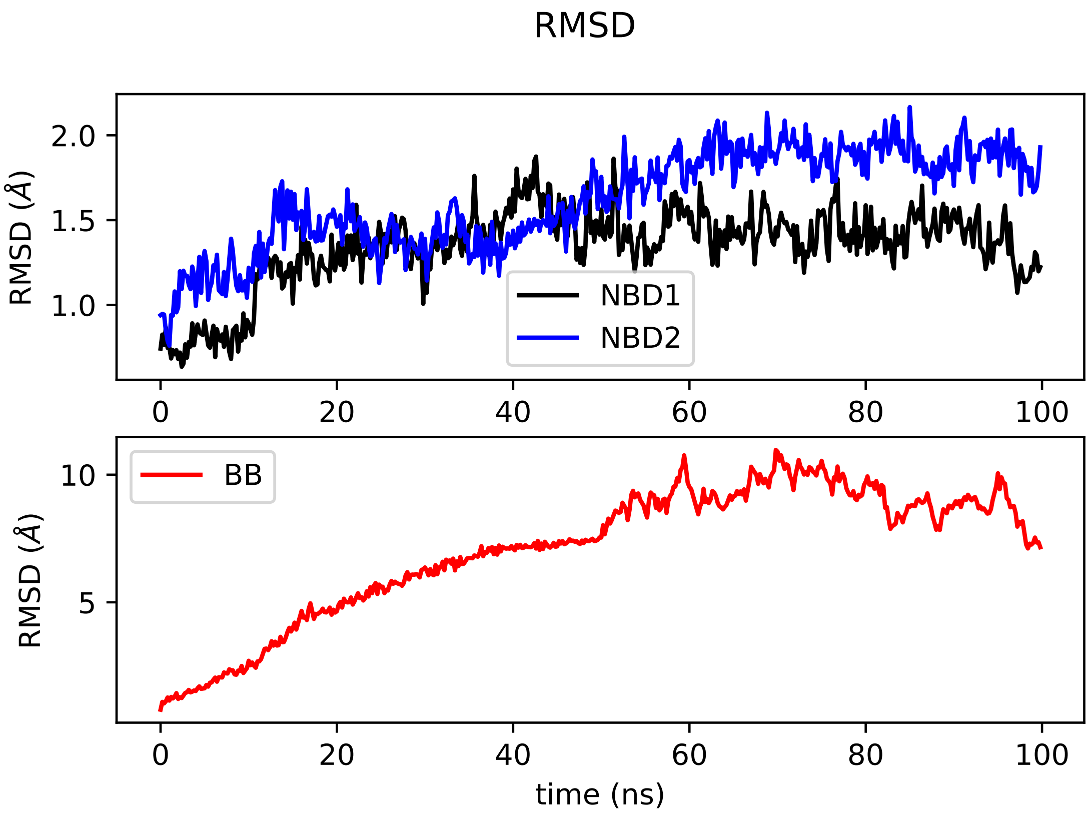

2 Table S1: Known P-gp nucleotide binding domain inhibitors

Structure	Name	Class	Ref.
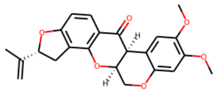	Rotenone	Active	[1]
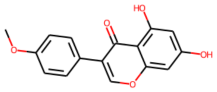	Biochanin A	Active	[2]
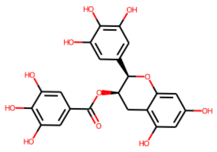	Epigallocatechingallate (EGCG)	Active	[3]
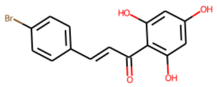	Compound 1	Active	[4]
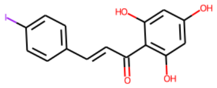	Compound 2	Active	[4]
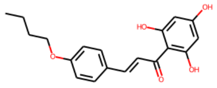	Compound 3	Active	[4]
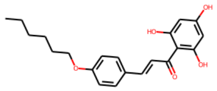	Compound 4	Active	[4]

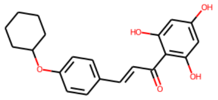	Compound 5	Active	[4]
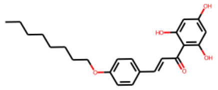	Compound 6	Active	[4]
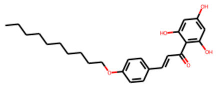	Compound 7	Active	[4]
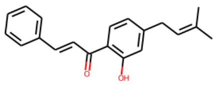	Compound 8	Active	[4]
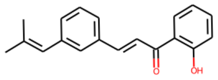	Compound 9	Active	[4]
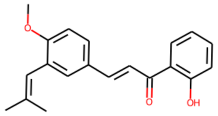	Compound 10	Active	[4]
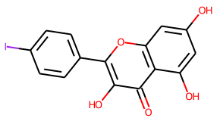	Compound 11	Active	[4]

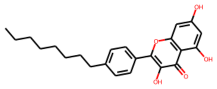	Compound 12	Active	[4]
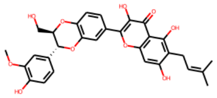	Compound 13	Active	[4]
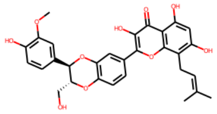	Compound 14	Active	[4]
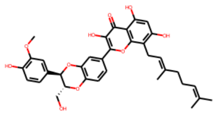	Compound 15	Active	[4]
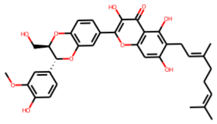	Compound 16	Active	[4]
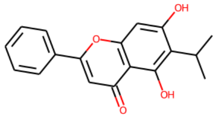	Compound 17	Active	[4]
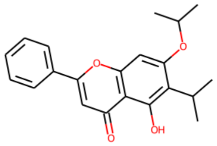	Compound 18	Active	[4]

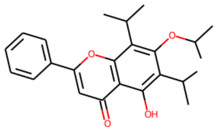	Compound 19	Active	[4]
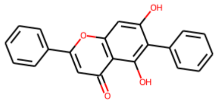	Compound 20	Active	[4]
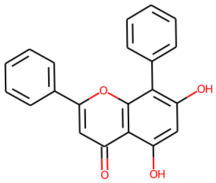	Compound 21	Active	[4]
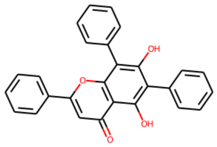	Compound 22	Active	[4]
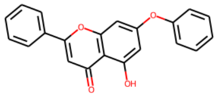	Compound 23	Active	[4]
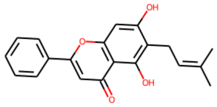	Compound 24	Active	[4]
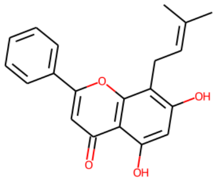	Compound 25	Active	[4]

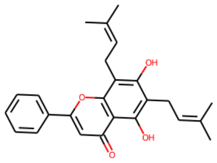	Compound 26	Active	[4]
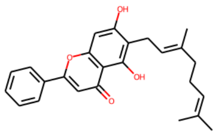	Compound 27	Active	[4]
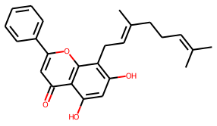	Compound 28	Active	[4]
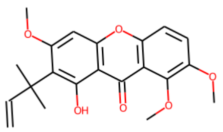	Compound 29	Active	[4]
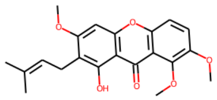	Compound 30	Active	[4]
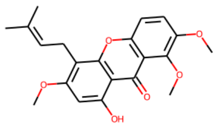	Compound 31	Active	[4]
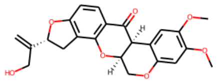	Amorphigenin	Marginal	[1]

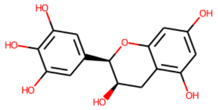	Epigallocatechin	Marginal	[1]
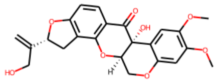	12a-Hydroxyamorphigenin	Marginal	[1]
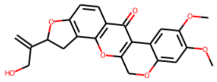	6a,12a,-Dehydroamorphigenin	Marginal	[1]
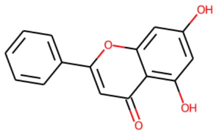	Chrysin	Marginal	[1]
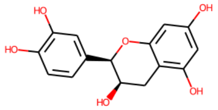	Epicatechin	Marginal	[5]
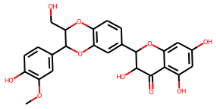	Silymarin	Marginal	[2]
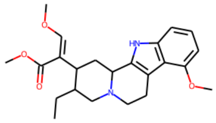	Mitragynine	Marginal	[6]

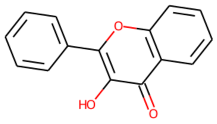	3OH-flavone	Marginal	[4]
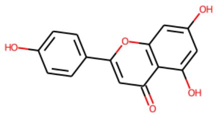	Apigenin	Marginal	[4]
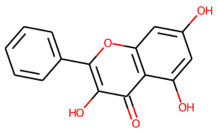	Galangin	Marginal	[4]
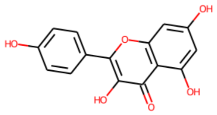	kaempferol	Marginal	[4]
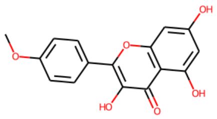	kaempferide	Marginal	[4]
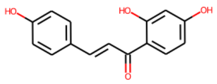	Compound 32	Marginal	[4]
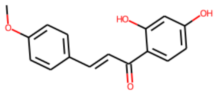	Compound 33	Marginal	[4]

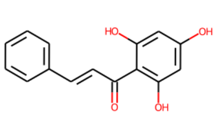	Compound 34	Marginal	[4]
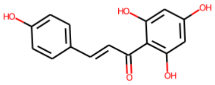	Compound 35	Marginal	[4]
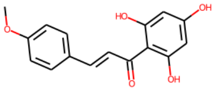	Compound 36	Marginal	[4]
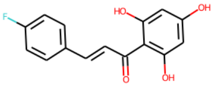	Compound 37	Marginal	[4]
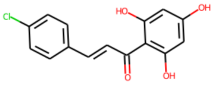	Compound 38	Marginal	[4]
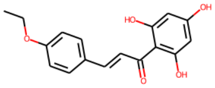	Compound 39	Marginal	[4]
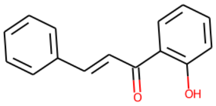	Compound 40	Marginal	[4]

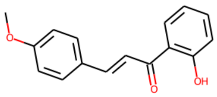	Compound 41	Marginal	[4]
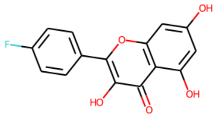	Compound 42	Marginal	[4]
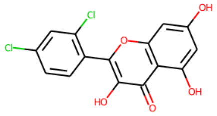	Compound 43	Marginal	[4]
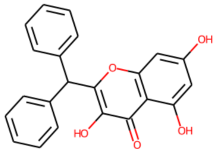	Compound 44	Marginal	[4]
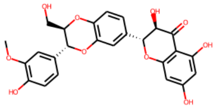	Silybin	Marginal	[4]
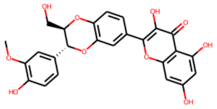	Dehydrodilybin	Marginal	[4]
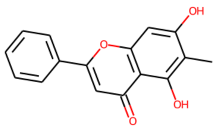	Compound 45	Marginal	[4]

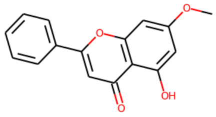	Compound 46	Marginal	[4]
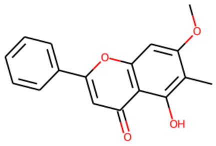	Compound 47	Marginal	[4]
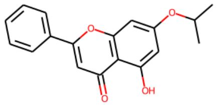	Compound 48	Marginal	[4]

3 Table S2: Results from retrospective dockings

	Time [ns]	NBD1 ATP RMSD	NBD1 Small set ROC/RIE	NBD1 Full set ROC/RIE	NBD2 ATP RMSD	NBD2 Small set ROC/RIE	NBD2 Full set ROC/RIE
No Mg^{+2}	0	1.8481	0.70/1.63	0.84/7.34	2.2647	0.70/1.61	0.78/3.22
	8	3.7874	0.61/1.29	0.72/2.84	2.0391	0.59/1.90	0.73/2.56
	20	4.2180	0.63/1.64	0.72/1.90	3.6227	0.76/4.62	0.84/6.38
	30	4.6506	0.65/1.17	0.74/2.04	3.4230	0.70/3.57	0.76/3.65
	50	3.5963	0.75/1.43	0.80/3.08	4.3758	0.80/5.03	0.82/5.91
	100	10.1041	0.65/2.73	0.76/3.13	4.6035	0.74/2.92	0.81/4.02
Mg^{+2}	0	1.9207	0.77/3.86	0.83/5.21	5.0070	0.81/4.24	0.84/5.07
	8	2.6346	0.73/3.57	0.78/4.21	1.9084	0.78/4.17	0.84/4.91
	20	3.7147	0.76/3.16	0.79/3.39	4.3953	0.77/4.27	0.81/4.19
	30	3.2729	0.68/2.60	0.74/3.01	3.9975	0.79/3.58	0.80/3.69
	50	4.4711	0.76/3.05	0.80/3.53	3.3475	0.75/3.01	0.78/3.18
	100	4.4711	0.76/3.05	0.80/3.53	3.3475	0.75/3.01	0.78/3.18

4 Fig. S2: Active learning Workflow

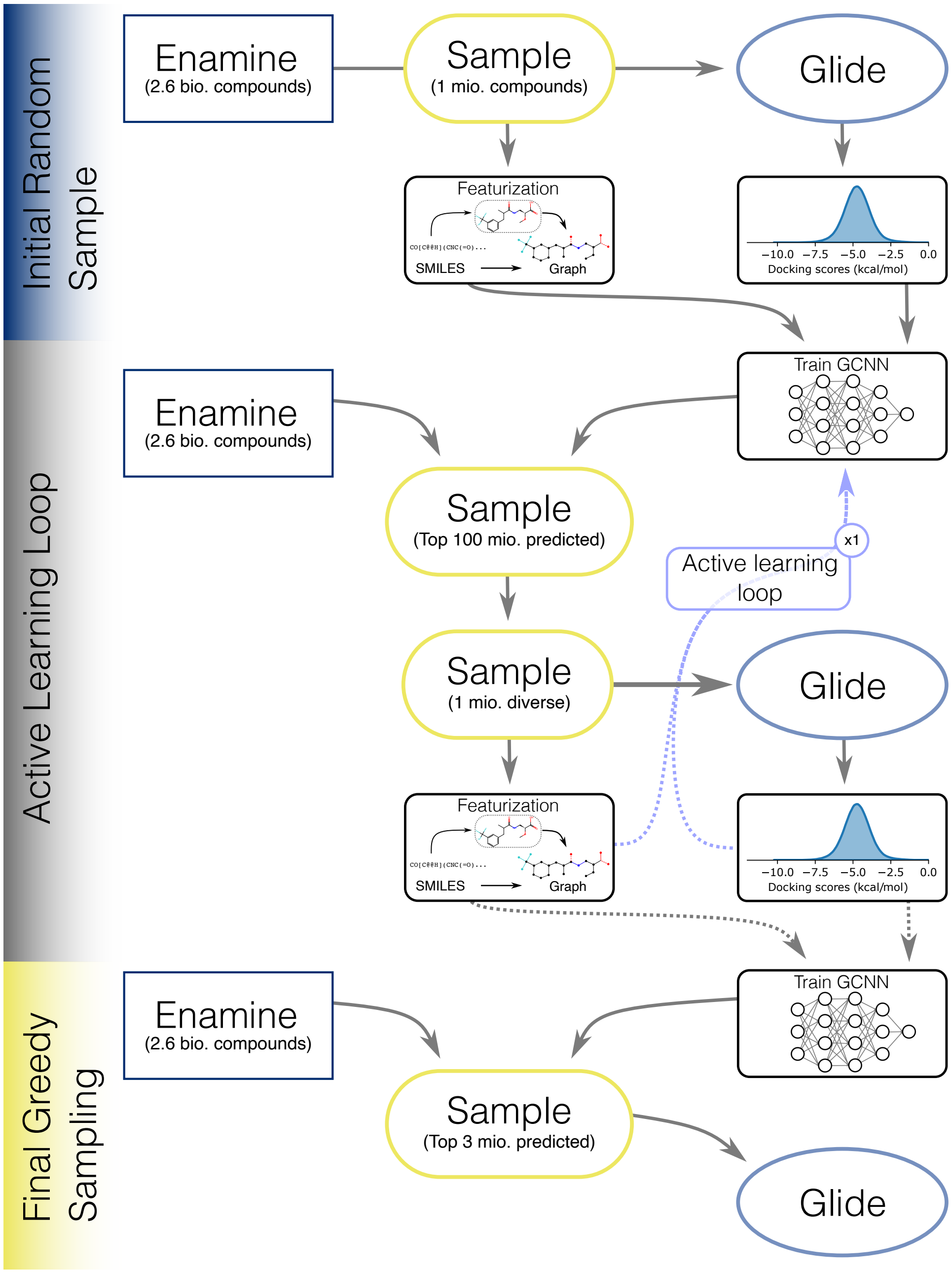

5 Fig. S3: Running average of the MM-GBSA score for the test simulations of P-gp bound to different ligands.

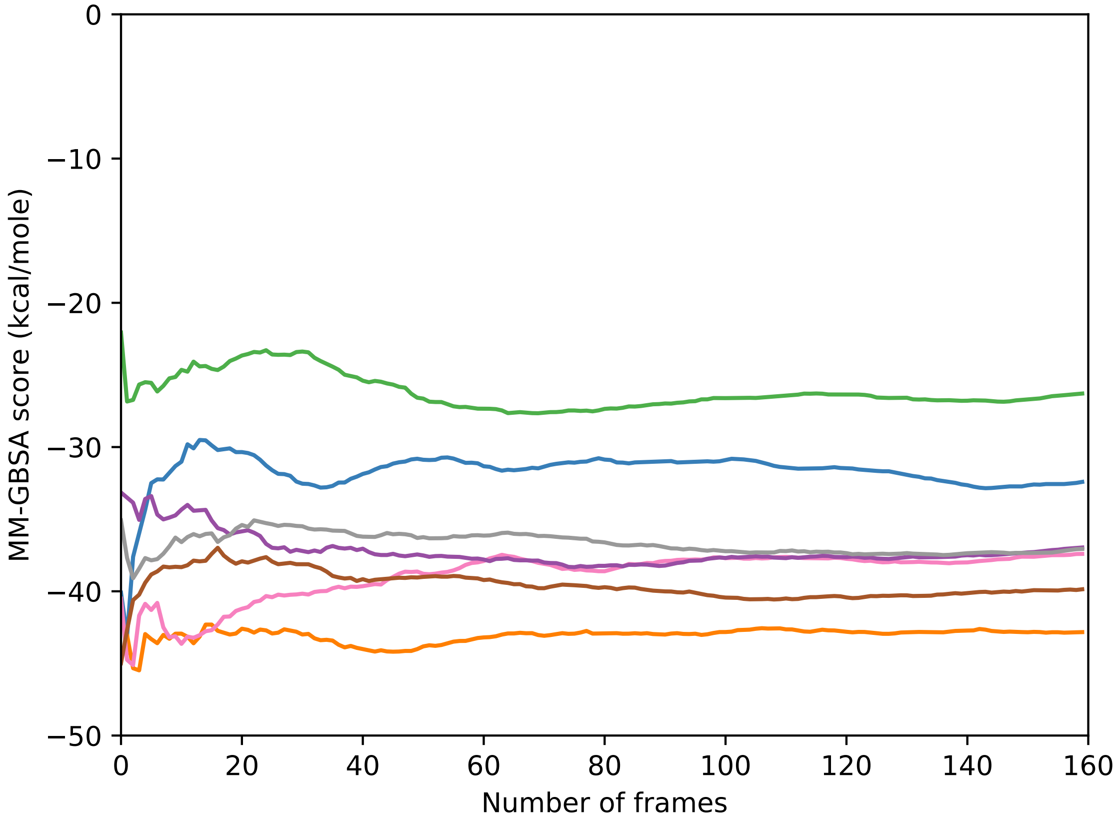

6 Fig. S4: Bootstrap estimation of docking score distribution of the full dataset

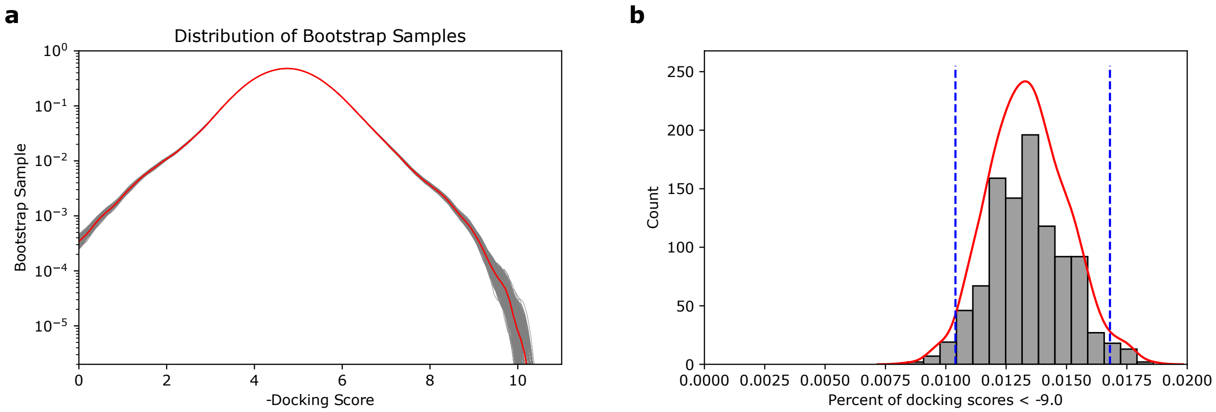

Fig. S4: a) Bootstrap estimates of the distributions of docking scores in the entire Enamine leadlike database. One thousand samples of 100,000 molecules were drawn from a pool of 1 million molecules. b) Histogram of the fraction of molecules in the bootstrapping samples with docking scores below -9 kcal/mol. Blue dashed lines: 95% confidence interval.

7 Fig. S5: Number of molecules belonging to the most populated scaffold for each round of the docking procedure.

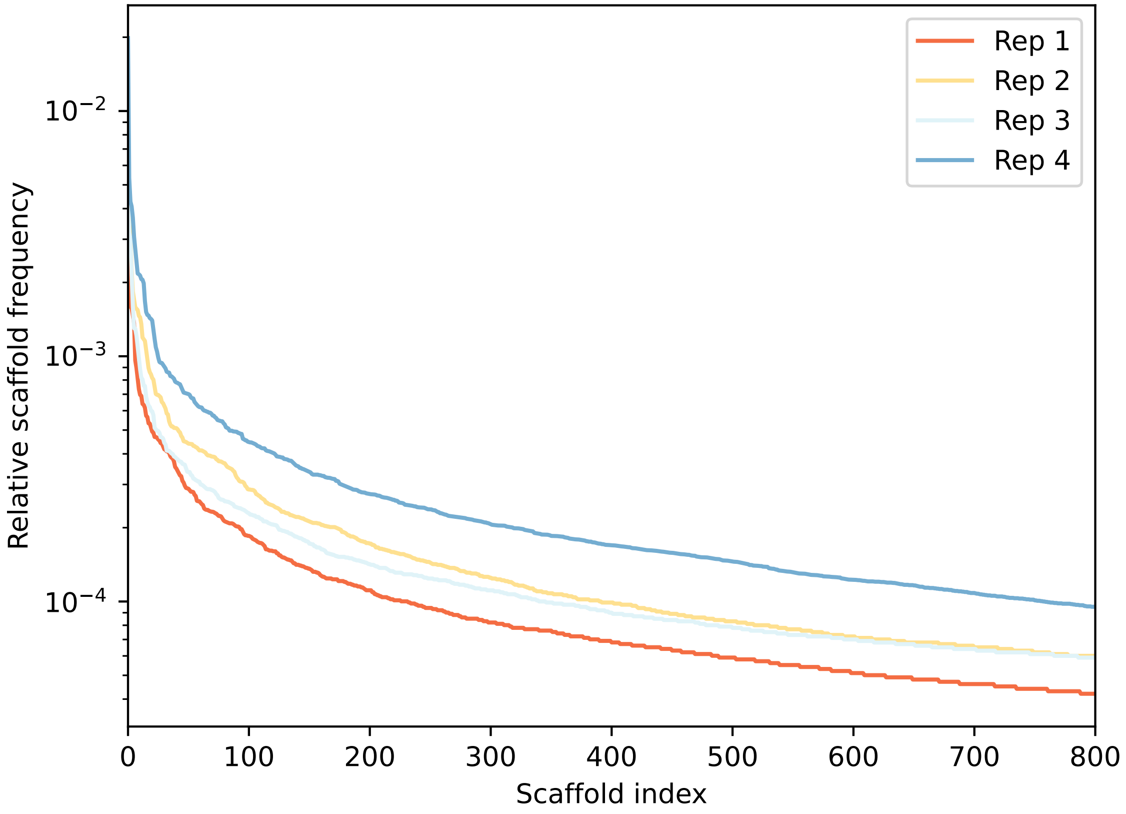

8 Fig. S6: Bemis-Murko scaffolds with best docking scores

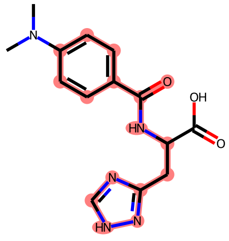

PV-002786790458

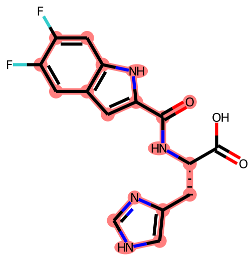

PV-002993852788

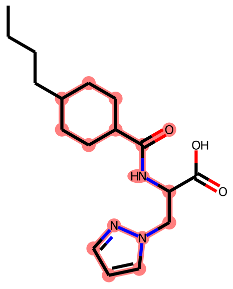

Z4246147137

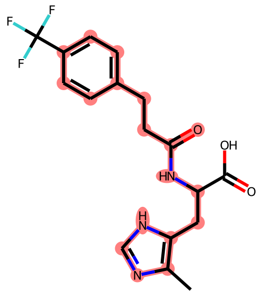

Z4420819076

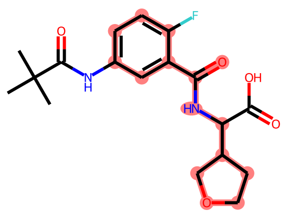

Z2656332796

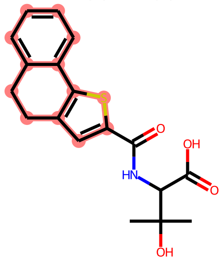

Z4248998813

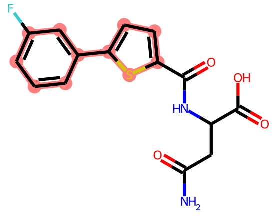

PV-002902001812

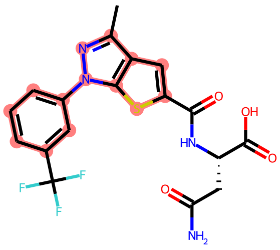

Z224156866

Fig. S6: Best scoring molecules that represent the most efficient Bemis-Murcko scaffold. Best scaffolds are defined as scaffolds contained by at least 100 docked molecules that have the best average score. The Bemis-Murcko scaffold is visualized by the red shades.

9 Fig. S7: MM-GBSA results

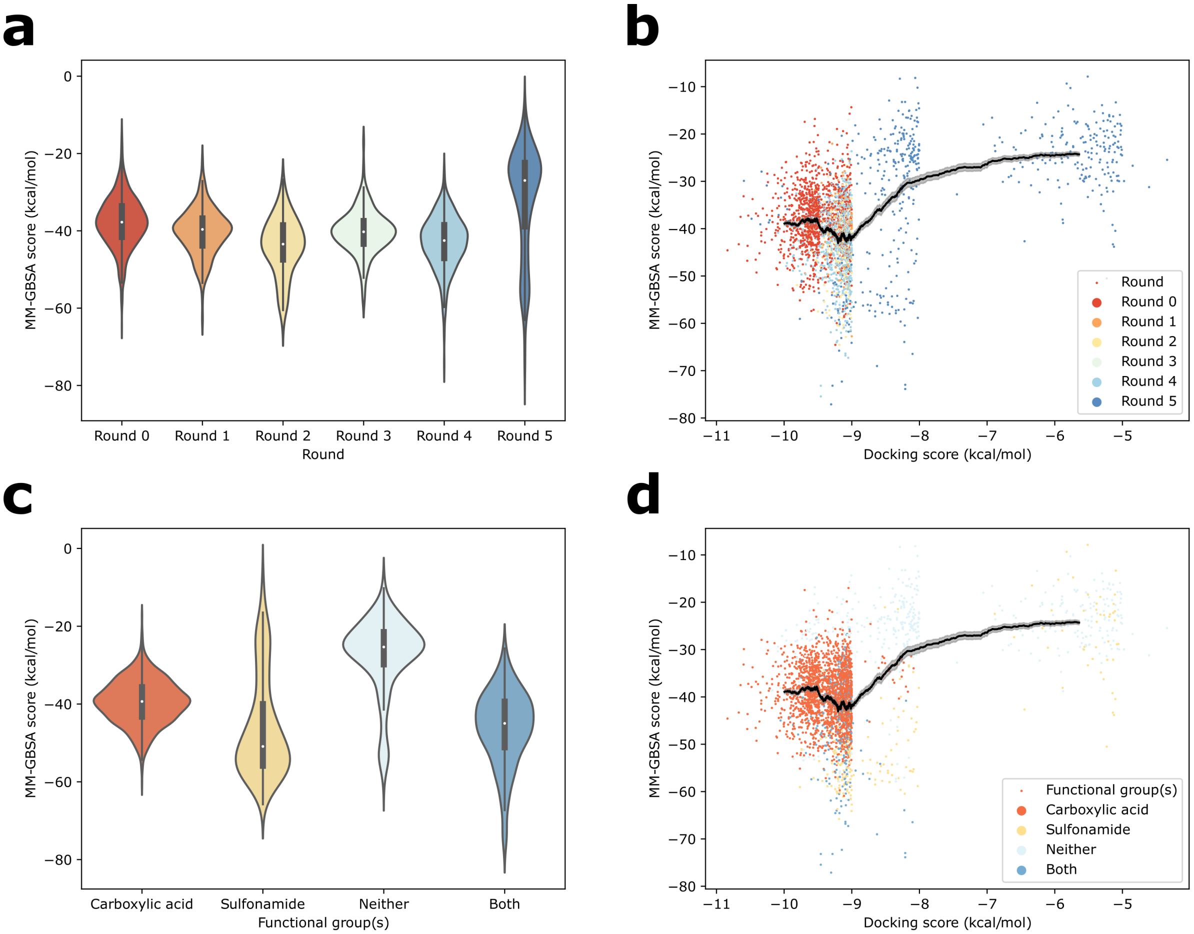

Fig. S7: Distribution of MM-GBSA scores according to docking scores across the MM-GBSA screening procedure. a) Violin plots of MM-GBSA scores from different rounds of the MM-GBSA scoring procedure. b) Plot of the MM-GBSA scores according to docking scores and round of MM-GBSA scoring. c) Violin plots of the MM-GBSA scores grouped according to the molecules' presence of carboxylic acids and sulfonamides. d) Plot of the MM-GBSA scores according to docking scores and the molecules' presence of carboxylic acids and sulfonamides.

10 Fig. S8: Experimentally tested compounds

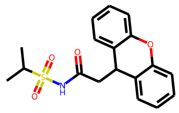

#01 (Z904895948)
Dscore: −8.03kcal/mol
MM-GBSA: −42.1kcal/mol

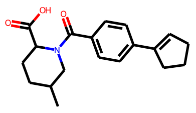

#02 (PV-004182297265)
Dscore: −9.08kcal/mol
MM-GBSA: −47.9kcal/mol

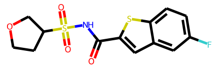

#03 (Z1197280865)
Dscore: −9.23kcal/mol
MM-GBSA: −51.8kcal/mol

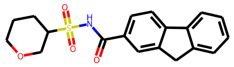

#04 (Z1630289915)
Dscore: −9.77kcal/mol
MM-GBSA: −59.1kcal/mol

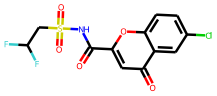

#05 (PV-003645777886)
Dscore: −8.31kcal/mol
MM-GBSA: −44.3kcal/mol

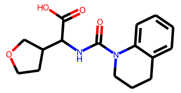

#07 (Z3568577220)
Dscore: −10.3kcal/mol
MM-GBSA: −37.2kcal/mol

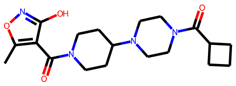

#08 (PV-005238982668)
Dscore: −9.19kcal/mol
MM-GBSA: −57.9kcal/mol

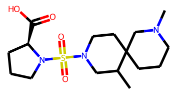

#09 (Z4341756354)
Dscore: −8.21kcal/mol
MM-GBSA: −73.9kcal/mol

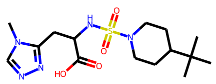

#10 (PV-004414630499)
Dscore: −9.05kcal/mol
MM-GBSA: −67.3kcal/mol

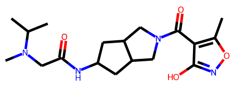

#11 (PV-003911020922)
Dscore: −9.08kcal/mol
MM-GBSA: −61.7kcal/mol

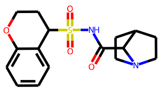

#12 (PV-002729996839)
Dscore: −8.02kcal/mol
MM-GBSA: −58.8kcal/mol

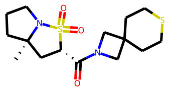

#13 (Z3610876513)
Dscore: −5.12kcal/mol
MM-GBSA: −43.8kcal/mol

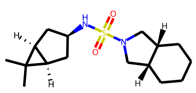

#14 (PV-002548537029)
Dscore: −5.23kcal/mol
MM-GBSA: −50.5kcal/mol

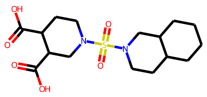

#15 (Z1739186218)
Dscore: −9.31kcal/mol
MM-GBSA: −77.1kcal/mol

11 Table S3: Overview of compounds and stock solutions

Catalog ID	paper ID	Purity (%)	MW	mg	µl	Solvent	mM
**Z904895948**	#01	90	345.4	10	200	DMSO	144.8
**PV-004182297265**	#02	90	313.0	10	200	DMSO	159.7
**Z1197280865**	#03	90	329.4	10	200	DMSO	151.8
**Z1630289915**	#04	90	357.4	10	200	DMSO	139.9
**PV-003645777886**	#05	90	352.0	10	200	DMSO	142.0
**Z3568577220**	#07	90	304.3	10	200	DMSO	164.3
**PV-005238982668**	#08	90	376.0	10	200	DMSO	133.0
**Z4341756354**	#09	90	359.5	10	700	DMSO	39.7
**PV-004414630499**	#10	90	373.0	10	200	DMSO	134.0
**PV-003911020922**	#11	90	364.0	10	200	DMSO	137.4
**PV-002729996839**	#12	90	336.0	10	600	DMSO	49.6
**Z3610876513**	#13	90	344.5	10	200	DMSO	145.1
**PV-002548537029**	#14	90	312.0	10	200	DMSO	160.3
**Z1739186218**	#15	90	374.5	9	200	DMSO	120.2

12 Fig. S9-11: Results from calcein-AM assay

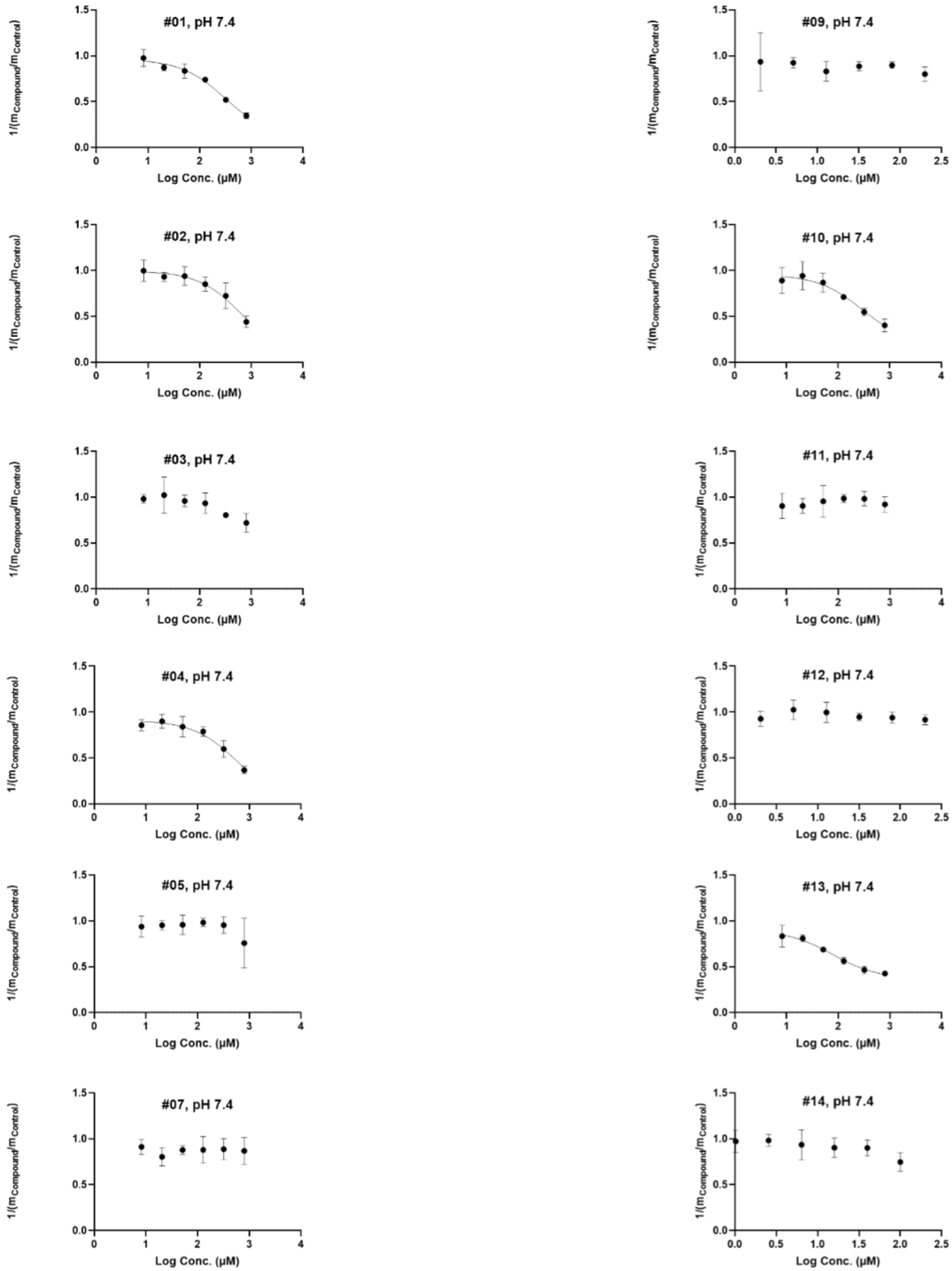

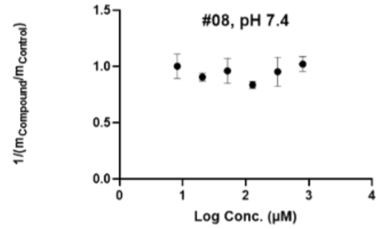

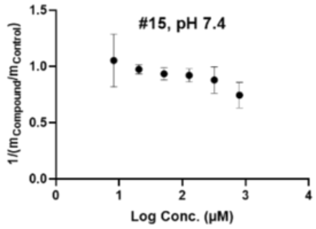

Fig S9: P-gp activity ($\left(1 \div \left[\frac{m_{compound}}{m_{control}}\right]\right)$) as a function of log concentration measured using the calcein AM assay at an extracellular pH of 7.4. For compounds that show inhibition of P-gp activity, IC50 values were estimated using Eq. 2, and the solid line is a fit of the data point to the equation. Data points are shown as mean $\pm$ SEM for three independent experiments (n = 3).

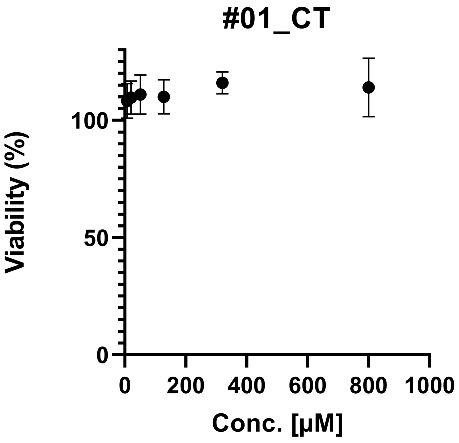
#01_CT
Viability (%)
0
50
100
0 200 400 600 800 1000
Conc. [µM]

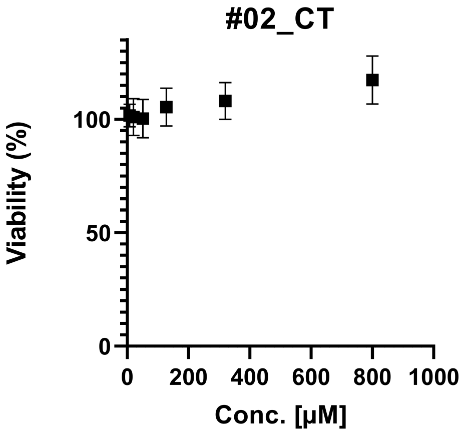
#02_CT
Viability (%)
0
50
100
0 200 400 600 800 1000
Conc. [µM]

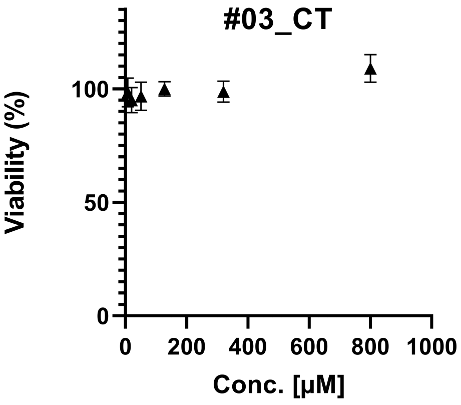
#03_CT
Viability (%)
0
50
100
0 200 400 600 800 1000
Conc. [µM]

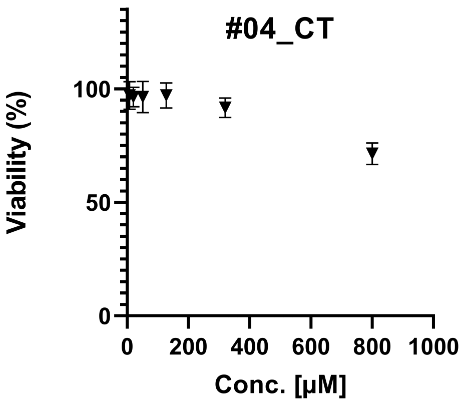
#04_CT
Viability (%)
0
50
100
0 200 400 600 800 1000
Conc. [µM]

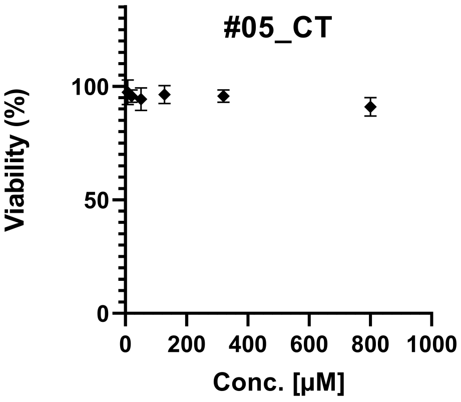
#05_CT
Viability (%)
0
50
100
0 200 400 600 800 1000
Conc. [µM]

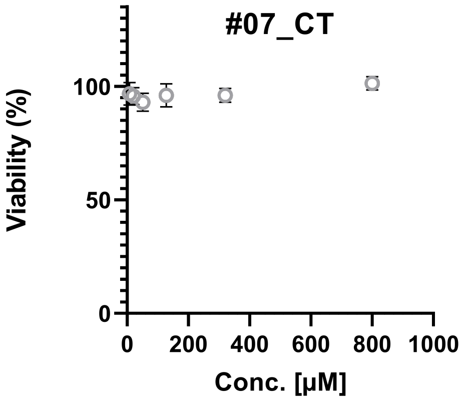
#07_CT
Viability (%)
0
50
100
0 200 400 600 800 1000
Conc. [µM]

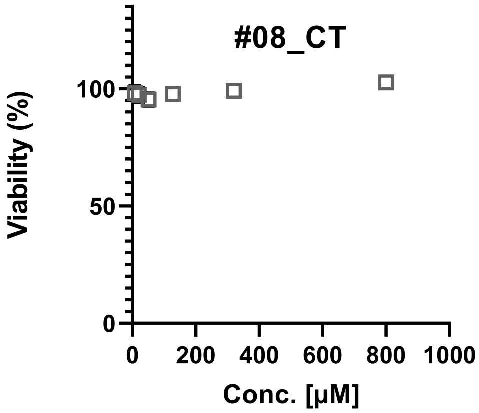
#08_CT
Viability (%)
0
50
100
0
200
400
600
800
1000
Conc. [µM]

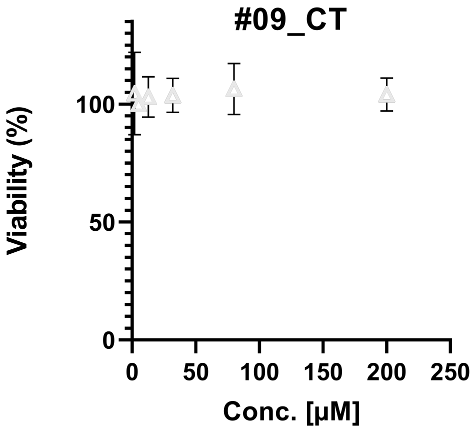
#09_CT
Viability (%)
0
50
100
0
50
100
150
200
250
Conc. [µM]

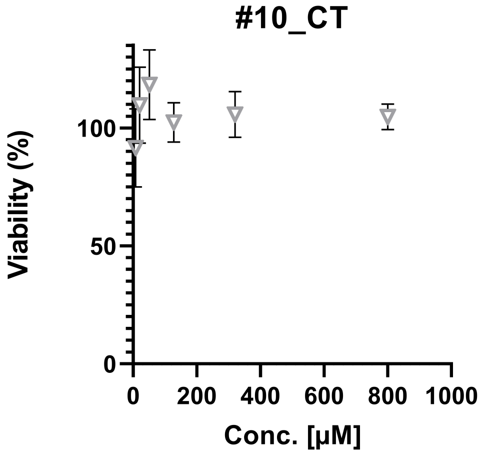
#10_CT
Viability (%)
0
50
100
0
200
400
600
800
1000
Conc. [µM]

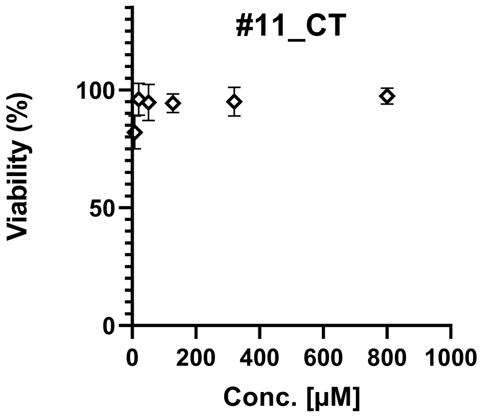
#11_CT
Viability (%)
0
50
100
0
200
400
600
800
1000
Conc. [µM]

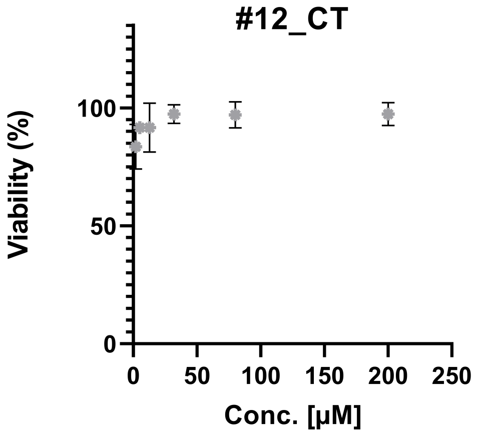
#12_CT
Viability (%)
0
50
100
0
50
100
150
200
250
Conc. [µM]

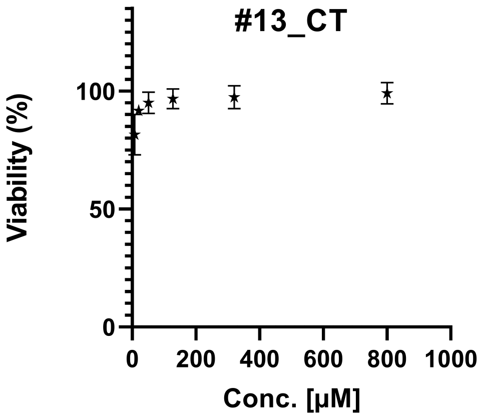
#13_CT
Viability (%)
0
50
100
0
200
400
600
800
1000
Conc. [µM]

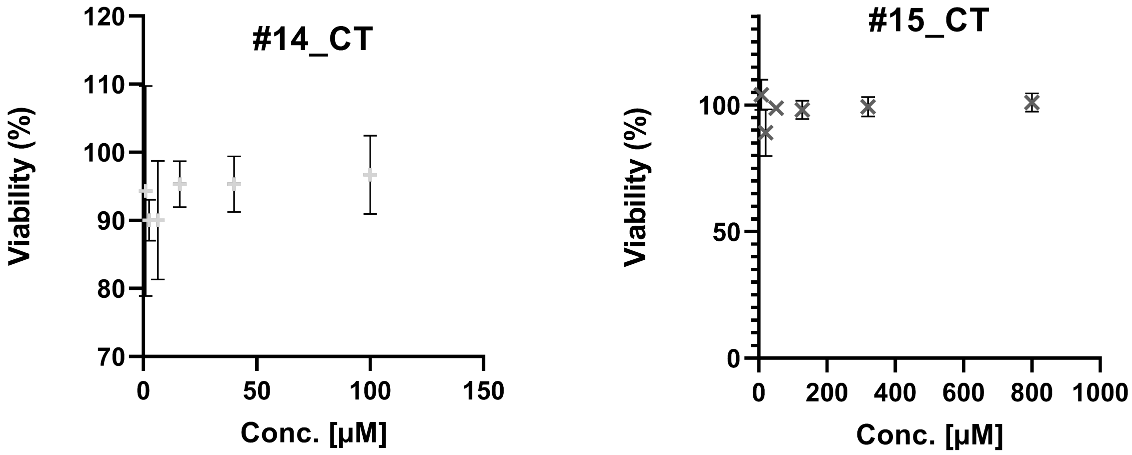

Fig S10: The cell viability of MDCKII MDR1 cells was evaluated after the calcein-AM assay using the Cell Titer Glow assay (CT), and the figure shows cell viability as a function of concentration. Bars shown are means ± SEM for three independent experiments (n = 3).

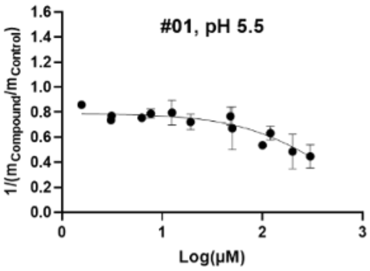
#01, pH 5.5
1/(mCompound/mControl)
Log(μM)

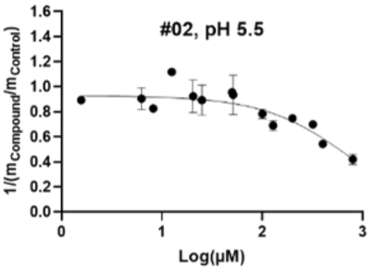
#02, pH 5.5
1/(mCompound/mControl)
Log(μM)

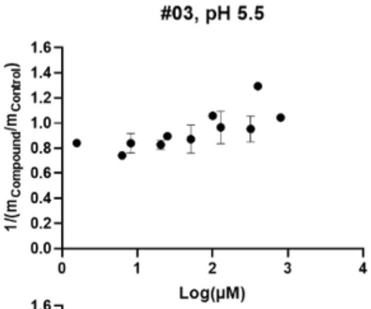
#03, pH 5.5
1/(mCompound/mControl)
Log(μM)

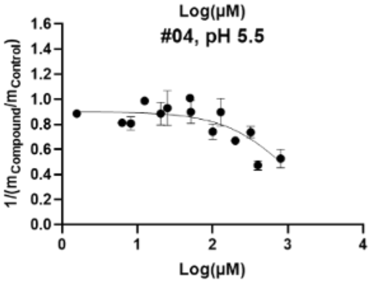
#04, pH 5.5
1/(mCompound/mControl)
Log(μM)

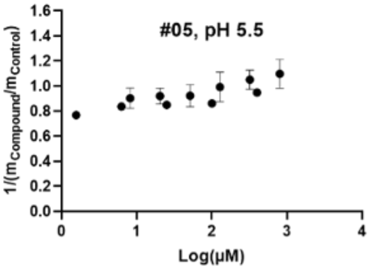
#05, pH 5.5
1/(mCompound/mControl)
Log(μM)

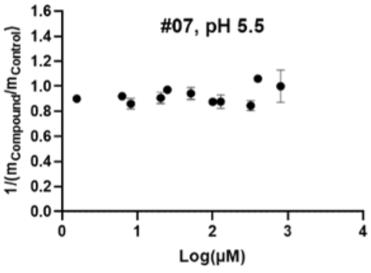
#07, pH 5.5
1/(mCompound/mControl)
Log(μM)

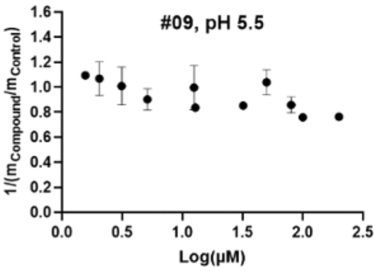
#09, pH 5.5
1/(mCompound/mControl)
Log(μM)

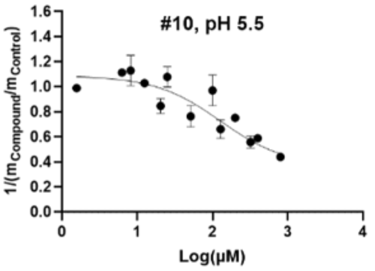
#10, pH 5.5
1/(mCompound/mControl)
Log(μM)

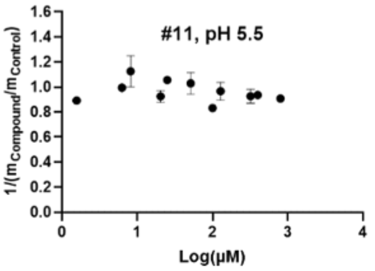
#11, pH 5.5
1/(mCompound/mControl)
Log(μM)

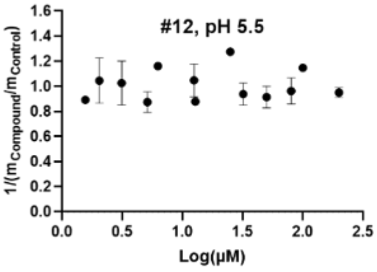
#12, pH 5.5
1/(mCompound/mControl)
Log(μM)

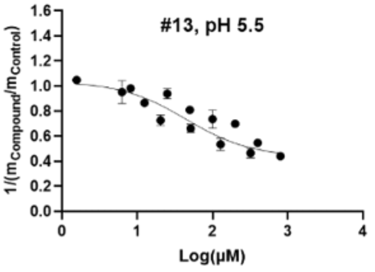
#13, pH 5.5
1/(mCompound/mControl)
Log(μM)

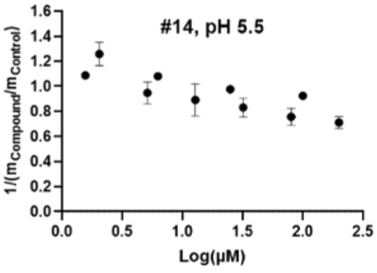
#14, pH 5.5
1/(mCompound/mControl)
Log(μM)

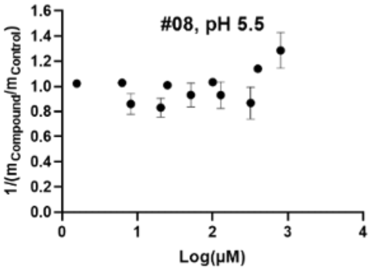

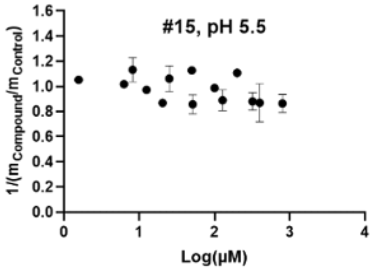

Fig S11: P-gp activity ($\left(1 \div \left[\frac{m_{compound}}{m_{control}}\right]\right)$) as a function of log concentration measured using the calcein AM assay at an extracellular pH of 5.5. For compounds that show inhibition of P-gp activity, IC50 values were estimated using Eq. 2, and the solid line is a fit of the data point to the equation. Data points are shown as mean ± SEM for three independent experiments (n = 3).

13 Fig. S12: Results from ATPase assay

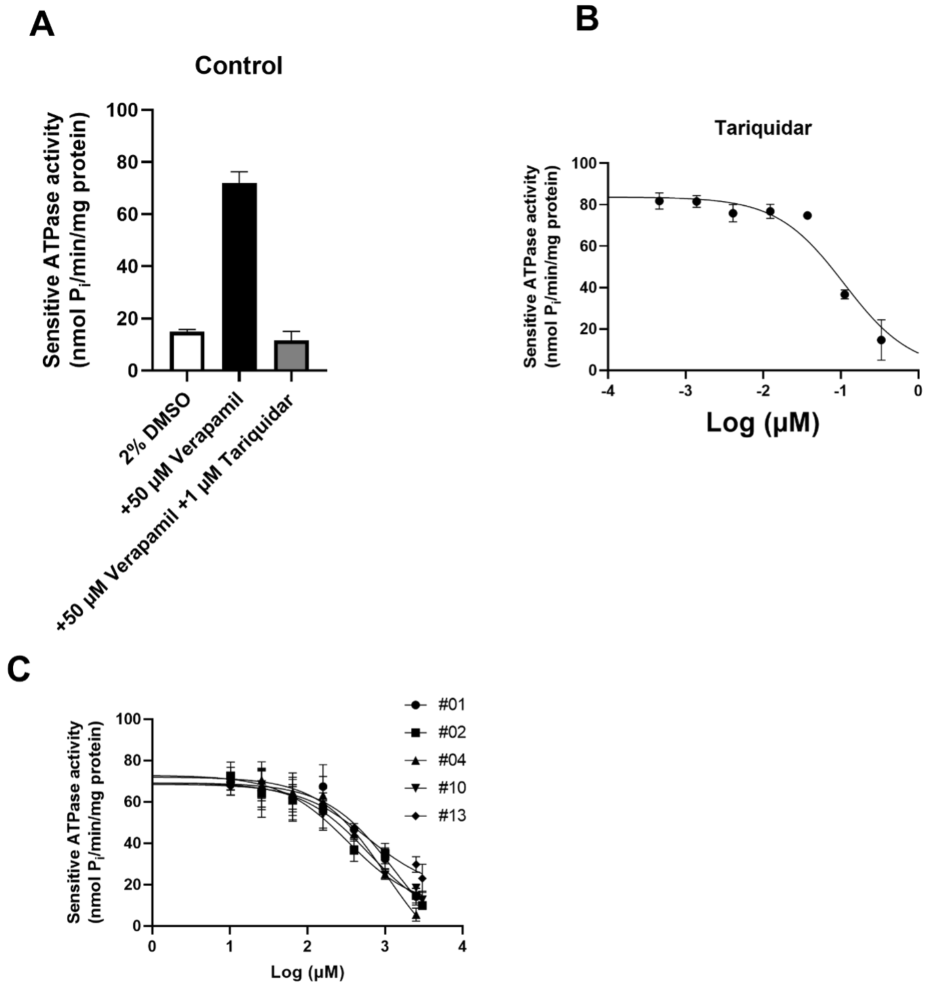

Fig S12: A) The amount of sensitive ATP used in P-gp expressing vesicles in buffer with either 2% DMSO, standard P-gp substrate 50 µM verapamil, or 1 µM Tariquidar plus verapamil. Data is mean and SEM from 8 experiments, n=8, for DMSO and verapamil, and n=6 for tariquidar. B) The amount of sensitive ATP used in P-gp expressing vesicles in buffer with 2% DMSO, 50 µM verapamil, and varying concentrations of Tariquidar. Data is mean and SEM from 3 experiments pr concentration level, n=3. C) The amount of sensitive ATP used in P-gp expressing vesicles in buffer with 2% DMSO, 50 µM verapamil, and varying concentrations of #1, 2, 4, 10, 13. Data is mean and SEM from 3 experiments pr concentration level, n=3.

14 Fig. S13: Comparing docking and MM-GBSA in predicting activity

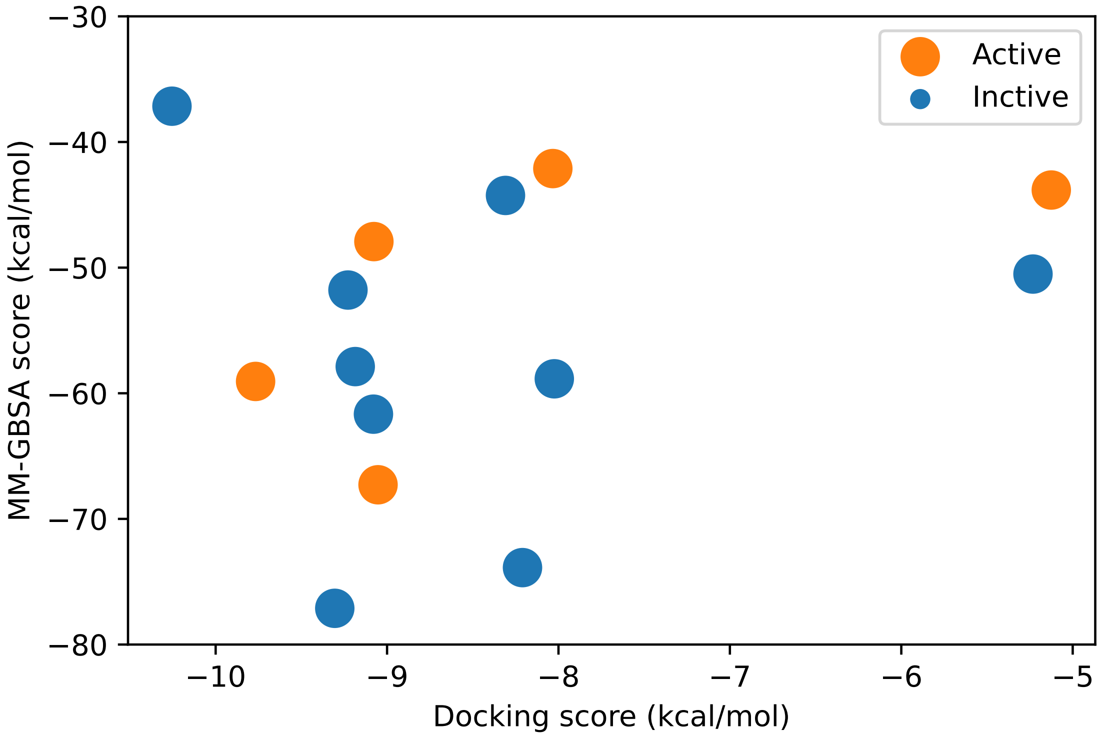

References

[1] Nóra Gyémánt, Masaru Tanaka, Sándor Antus, Judit Hohmann, Orsolya Csuka, L Mandoky, and J Molnar. In vitro search for synergy between flavonoids and epirubicin on multidrug-resistant cancer cells. *In vivo*, 19(2):367–374, 2005.

[2] Shuzhong Zhang and Marilyn E Morris. Effects of the flavonoids biochanin a, morin, phloretin, and silymarin on p-glycoprotein-mediated transport. *Journal of Pharmacology and Experimental Therapeutics*, 304(3):1258–1267, 2003.

[3] Shuji Kitagawa, Tomohiro Nabekura, and Shizu Kamiyama. Inhibition of p-glycoprotein function by tea catechins in kb-c2 cells. *Journal of Pharmacy and Pharmacology*, 56(8):1001–1005, 2004.

[4] Ahcene Boumendjel, Attilio Di Pietro, Charles Dumontet, and Denis Barron. Recent advances in the discovery of flavonoids and analogs with high-affinity binding to p-glycoprotein responsible for cancer cell multidrug resistance. *Medicinal research reviews*, 22(5):512–529, 2002.

[5] A Martins, A Vasas, ZS Schelz, M Viveiros, J Molnar, J Hohmann, and L Amaral. Constituents of carpobrotus edulis inhibit p-glycoprotein of mdr1-transfected mouse lymphoma cells. *Anticancer research*, 30(3):829–835, 2010.

[6] Noradliyanti Rusli, Azimah Amanah, Gurjeet Kaur, Mohd Ilham Adenan, Shaida Fariza Sulaiman, Habibah Abdul Wahab, and Mei Lan Tan. The inhibitory effects of mitragynine on p-glycoprotein in vitro. *Naunyn-Schmiedeberg's archives of pharmacology*, 392(4):481–496, 2019.
